# Supplementary material for: Are carbon emissions trading and green financial instruments synergistic? -Comprehensive quantitative research based on content analysis
Source: PLoS One. 2024 Mar 7;19(3):e0298601. doi: 10.1371/journal.pone.0298601 (PMC10919601; doi:10.1371/journal.pone.0298601)
Supplement: S1 Data — (ZIP) [file pone.0298601.s001.zip › Data/地级市Z_F工作报告.zip!/╣π╢1⁄2/╒┐╜¡╩╨/└┤╫╘╣1⁄2╓┌║┼í╛┬φ┐╦╩2╛▌═°í┐_2009.docx]

各位代表：

　　我代表市人民政府向大会作政府工作报告，请予审议，并请各位政协委员和其他列席人员提出意见。

　　2008年工作回顾

　　过去的一年，受外部严峻复杂经济环境特别是国际金融危机和罕见低温冰冻灾害的不利影响，我市经历了一系列新困难新挑战。在省委、省政府和中共湛江市委的正确领导下，在市人大及其常委会和市政协的监督支持下，市政府认真贯彻党的十七大和十七届三中全会精神，全面落实科学发展观，围绕建设城乡协调、生态文明的科学发展试点市，积极实施“工业立市、港口兴市、生态建市”发展战略，团结一心，迎难而上，艰苦拼搏，狠抓落实，经济社会保持平稳较快发展，完成了市十二届人大三次会议确定的目标任务，各项工作取得新的成就。

　　经济总量首次登上千亿元台阶。全市完成生产总值1048.66亿元，增长10%。其中，第一产业增加值230.66亿元，增长5.4%；第二产业增加值497.07亿元，增长10.5%；第三产业增加值320.93亿元，增长12.5%。人均生产总值15297元，增长8.4%。单位生产总值能耗下降2.75%，降幅居全省第二。地方财政一般预算收入45.7亿元，增长19.5%，人均财政收入666元，增长18%；来源于湛江的财政总收入292亿元，增长57.84%。

　　工业经济增势喜人。全市规模以上工业总产值首次突破千亿元，达到1131.65亿元，增长13.5%；增加值393.45亿元，增长11.2%。工业经济效益综合指数288.5%，居全省第二位。湛江钢铁项目获得国家批准开展前期工作，配套设施建设和征地拆迁工作全面铺开；中海油南海西部油田实现油气当量1000万立方；东兴石油公司和中海油湛江分公司成为年销售收入超200亿元企业；东兴炼油500万吨配套完善、龙腾物流500万吨球团等项目顺利推进，70万吨木浆等项目开工；湛江经济技术开发区东海岛新区建设进展顺利。工业产品新增名牌19个；吴川羽绒产业集群升级示范区获省认定。工业对经济增长的贡献率为46.5%，成为经济增长的强劲动力。

　　港口发展实现新跨越。全市港口货物吞吐量首次突破亿吨，达到1.04亿吨，增长13.5%，迈进全国15个亿吨大港行列，成为我国西南沿海港口群唯一的亿吨大港，进一步确立了大西南出海主通道的优势和地位。其中，湛江港（集团）股份有限公司完成货物吞吐量5956万吨，增长11.4%。

　　农村经济总量保持全省第一。实现农业总产值380亿元，增长5.4%。新增重点农业龙头企业国家级2家、省级6家，建设特色专业镇4个，市级以上农业龙头企业年销售收入130多亿元，带动35万农户平均每户增收3200多元。农业机械化水平全省领先，农业节水技术在全国推广。被列为国家农村信息化综合信息服务试点市，成功举办广东省农村信息化成果展。海峡两岸农业合作试验区新增台资企业5家。造林24.6万亩，森林覆盖率27.7%；吴川市成为我市第4个省级林业生态县（市）。

　　“双转移”工作取得新成效。承接珠三角产业转移单个项目14项，累计达到185项，总投资435.7亿元。成功承办省第五届“山洽会”，我市签约19个项目，签约总额117.58亿元。顺德（廉江）、龙岗（吴川）产业转移工业园建设取得新进展，广州（湛江）产业转移示范性工业园筹建顺利。组织农村劳动力培训5.5万人，转移就业22万人。

　　基础设施和城市建设有力推进。完成固定资产投资295.29亿元、增长21.6%，其中重点项目完成投资61.3亿元。建成黎湛铁路河唇至湛江段增建二线、湛江港30万吨级航道、海安新港一期等项目；加快建设湛江港宝满集装箱码头；动工建设国道325线文车至北罗坑一级公路和南三大桥；鉴江供水枢纽、茂湛铁路、湛徐高速公路等项目前期工作进展顺利。编制完成坡头海东新区、赤坎海田片区和麻章区控制性详细规划，启动编制南三岛分区规划。市区建成体育南路、体育北路东段、百蓬路等一批骨干道路和南国热带花园，新增城市绿地100.2万平方米，绿地率40.65%，绿化覆盖率45.71%，人均公园绿地面积12.51平方米。霞山污水处理厂一期工程建成投产，赤坎水质净化厂二期工程和吴川污水处理厂顺利推进；关闭10家产能落后和污染严重企业；100辆油改气公交车、300辆油气双用燃料出租车投入营运，新增和改造市区108个港湾式候车亭。市区生活垃圾无害化处理率93%以上。城市环境空气质量继续保持全国重点考核城市前列、全省首位。农村基础设施不断改善，建成镇村公路硬底化1269公里、乡镇客运站和农村客运站场各10个、客运候车亭656个，客运通达率85%；实施城乡水利防灾减灾工程31宗，完成省人大议案督办的小型水库除险加固工程335宗。完成830个新农村建设规划编制。徐闻县曲界镇和廉江市河唇镇被命名为省级生态示范镇。

　　消费拉动能力显著增强。社会消费品零售总额467.7亿元，增长22.4%，增速创1996年来新高。建成嘉信茂沃尔玛、兴华海之城和国贸新天地数码城三大商场和一批高等级酒店。5个县（市）、6个企业成为全国“万村千乡市场工程”试点单位，建成或改造配送中心10家、农家店1738个。全市金融机构储蓄存款余额首次突破千亿元，达到1075.8亿元，增长16.1%；贷款余额476.9亿元，增长20.3%。接待游客891万人次，增长15.9%；旅游业总收入47亿元，增长13.9%。

　　体制改革不断深化。建立财政支出项目事前申报绩效目标机制，行政事业单位国有资产有偿使用收入全部纳入预算统一管理，财政资金管理实现全程联网，开展公务卡结算和公务定点接待制度改革试点。推进部门业务电子化集中监管，清理压减行政许可项目263个，行政审批实际办结比法定时限平均缩短17.4个工作日。全面实施国有资产收益收缴制度，上缴收益2.76亿元。湛江港（集团）股份有限公司、湛化股份公司、湛汽集团公司等企业通过战略合作、合资并购勃发生机活力；44家劣势企业平稳退出市场，筹资1.54亿元，安置职工4108人；国有粮食企业改革全面完成。市商业银行实现经营利润4.49亿元，获准筹建广州分行，荣获“2008年中国最具成长力的金融机构”称号；农村信用社改革稳步推进，获得11.79亿元央行票据兑付。在全省率先完成县（市）、镇两级供电企业体制改革，雷州青年运河管理局和9个县（市、区）完成水利管理体制改革。民营经济增加值464.75亿元，增长15.1%，占全市生产总值44.3%，对经济增长贡献率达60%。

　　外经贸逆势上扬。积极应对国际形势新变化，对外开放进一步扩大。实际吸收外资1.73亿美元，增长15.4%；外贸进出口33.29亿美元、增长29.3%，其中出口16.32亿美元、增长11.7%，进口16.97亿美元、增长52.3%。海关、检验检疫等口岸部门积极支持外经贸工作，扶持农海产品扩大出口。对外友好合作进一步加强。

　　社会事业全面进步。实施科技计划项目450个，取得科技成果66项，23家企业被认定为高新技术企业，高新技术产品总产值增长8%；建设4个国家级、3个省级农业标准化示范区；建成省级企业技术中心1个，组建市级工程中心、技术开发中心5个，建设农业科技示范基地6个，新增国家级地理标志保护产品1个。全面实施城乡免费义务教育，直接减轻群众负担5.56亿元；新建校舍1112幢，新增高中学位3万多个，高中阶段教育毛入学率65%，比上年提高11个百分点；高考本科以上上线人数高于全省平均增幅一倍。有效防控重大疾病和突发公共卫生事件；市一中医院被列入国家重点中医院建设单位。湛江籍运动员在国内外大赛中累计成绩排名全省第四位，参加北京奥运会和残奥会获得4枚奖牌，其中何冲夺得奥运会跳水比赛男子单人3米跳板冠军，符洪芝夺得残奥会女子坐姿反曲弓比赛金牌。成功举办市第七届艺术节和北部湾首届动漫节，湛江粤剧院筹建工作加快，启动“农家书屋”建设工程；300多件文艺作品获国家、省奖励；非物质文化遗产保护工作卓有成效；新闻媒体、湛江书城实现文化经营收入超亿元。人口出生率11.5‰，自然增长率6.98‰，实现县（市、区）计生工作达标。顺利完成村委会和社区换届选举工作。119个基层武装部“四个基本”建设全面达标；我市第三次被命名为全国双拥模范城、第四次被命名为省双拥模范城，遂溪县、徐闻县、廉江市分别被命名为省双拥模范县。对口援建四川省汶川县龙溪乡恢复重建，4个项目动工建设，其中镇村供水项目建成。不断完善应急管理体系，制定青少年地震演练和地震知识普及实施计划。加强审计监督，规范财政资金管理使用。加大价格调控力度，物价总水平基本保持稳定。气象部门积极提供天气预报服务。统计、外事、侨务、人事、编制、地方志、档案、国家安全等工作得到加强，人防、海防、打私、民兵预备役、民族宗教、妇女儿童、残疾人、老龄等工作取得新成绩。

　　人民群众享有更多实惠。全年用于社会民生领域的财政投入67.59亿元，占地方财政一般预算支出的62.1%，是社会民生投入力度最大、城乡居民受益最多的一年。城镇居民人均可支配收入12362元、增长8.5%，农民人均纯收入5330元、增长7%。城镇新增就业5.36万人，下岗失业人员再就业4.2万人；残疾人就业率85%以上。企业基本养老保险新增31907人，失业保险新增26875人，职工医疗保险新增21100人，工伤保险新增41230人。农村年人均纯收入低于1200元的困难群众实现应保尽保。在全省欠发达地区率先推行城乡基本医疗保险一体化，城乡居民参保563万人，其中城镇居民参保67万人、参保率82.84%，农村居民参保496万人、参保率96.25%。建设廉租房16562平方米、安置房3986平方米。完成2万户农村贫困户危房、茅草房改造；扶持在去年强台风中房屋全倒的2682户村民兴建新房；建设水库移民新村93个，解决4300户、1.8万人的住房困难；建成153户水上居民和贫困渔民安置住房。有效解决43.35万农村人口饮水安全问题。提高农村五保供养和城乡低保对象最低生活保障标准，农村“两委”干部补贴每人每月增加100元。行政村药品供应和监督网实现全覆盖。实行老年人免费乘坐公交车，发放免费乘车IC卡12980张。

　　精神文明和民主法制建设进一步加强。广泛开展群众性精神文明创建活动，市地税局被评为全国文明单位，市房产管理局等5个单位被评为全国精神文明建设工作先进单位；创建市级生态文明村225个，初步建成10个生态文明区（片、带），吴川市蛤岭村、徐闻县广安村被评为全国文明村，霞山区特呈岛坡尾村、麻章区大路前村被评为全国创建文明村镇先进单位。办理省、市人大代表议案143件和省、市政协委员提案193件，满意率均达97%。建成行风热线网、行政审批电子监察网、手机信访系统和村务信息公开网，形成网络监督系列平台。政务公开示范单位试点工作顺利推进。深入开展安全“隐患治理年”活动，全面实现省下达的安全生产控制考核目标。继续开展产品质量和食品安全专项整治。35个社区被省命名为“六好”平安和谐社区；公安“三基”工程建设通过省公安厅考核验收，刑事、治安案件和群体上访事件整体下降，公众安全感满意率进一步提高。畅通诉求渠道，规范信访秩序，信访量下降16%。组建农村维稳律师团，扩大法律援助覆盖面。

　　各位代表，回顾2008年，我市在复杂多变的国内外形势和历史罕见的困难与挑战面前，经济社会发展取得这样好的成绩，确实来之不易，这是我们认真贯彻落实科学发展观的结果，是中共湛江市委正确领导，市人大、市政协监督支持的结果，是全市人民群策群力、艰苦奋斗的结果。我代表市人民政府，向各级人大代表和政协委员，向全市广大工人、农民、知识分子、干部，向离退休老同志，向驻湛人民解放军、武警官兵、人民警察和各民主党派、人民团体、社会各界人士，向关心和支持湛江发展的港澳台同胞、海外侨胞、国际友人，表示崇高的敬意和衷心的感谢!

　　在充分肯定成绩的同时，我们也清醒地认识到，我市经济社会发展还存在不少困难和问题，主要表现在：总体经济规模仍然偏小，发展速度不够快，尤其是受宏观经济环境变化的影响，部分主要经济指标增速出现回落，一些经济实体生产经营困难加剧，促增长、保发展的压力增大；支柱产业不够强，高新技术产业、现代服务业发展相对滞后，加快调整优化产业结构更加紧迫；工业化、城镇化水平仍然不高，以工促农、以城带乡能力不强，缩小发展差距和促进协调发展任务还很艰巨；影响科学发展的体制机制瓶颈尚未突破，尤其是运用市场机制破解发展难题、推进创新发展的动力不足、能力不强，改革攻坚任务还很繁重；对外开放整体水平不高，加上国际金融危机的影响，出口和吸收外资将面临更多困难；城乡规划执行不够有力，旧城区和城中村改造进展缓慢，城市管理中还存在一些急需解决的顽症，违法违规建设问题比较突出，城市建设和管理水平有待进一步提升；社会民生领域仍然投入不足，教育、医疗、就业、社保、住房等关系群众切身利益的民生建设需要进一步加强。政府自身建设与推动科学发展的要求还不完全适应，一些部门和干部思想不够解放、创新进取的锐气不足，缺乏攻坚克难、敢于负责的精神；一些部门为企业、为市民服务的主动性不高，办事难现象仍然存在，“文山会海”问题还未根本解决等等。对这些问题，我们务必增强危机感、责任感和紧迫感，采取更加有力措施认真解决。

　　2009年工作安排

　　2009年是新中国成立六十周年，也是我市推进科学发展、实现“十一五”规划目标、建设全面小康社会非常关键的一年。综观国际国内形势，国际金融危机尚未见底，国内外经济增长明显减缓。我们既要充分认识国际金融危机带来的困难，把应对措施考虑得周密一些，又要科学判断危机对我市的影响，抓住危机所蕴含的前所未有的机遇，正如中央政治局委员、省委书记汪洋同志在省人大会议期间参加湛江代表团讨论时指出：“金融危机对湛江影响较小，机遇更多，湛江要倍加努力，逆势崛起”。我们要进一步贯彻落实好汪洋书记的指示精神，采取积极有为的应对措施，努力实现逆势崛起。去年底以来，国家和省为应对国际金融危机陆续出台一系列保增长、扩内需、调结构的政策举措，我市当前开工或筹建的一批重大项目，顺应这些政策而成为国家和省增加投资的重点，从今年起到2012年，全市将实施八大重点工程、81个重点项目，总投资2161亿元，无论是项目数量还是投资力度，都是我市历史上空前的，这是我们有效应对危机、加快发展的有力保障；随着国务院批准的《珠江三角洲地区改革发展规划纲要》的深入实施，省里对湛江更加关注和大力支持，将为新一轮发展注入强大动力；我市在土地、劳动力、生态环境等方面的后发优势也将更加凸显，成为吸引项目和投资的优越条件。可以说，我市具有化“危”为“机”、战胜困难的独特优势。只要我们审时度势、沉着应对、抢抓机遇、共克时艰，就能变危机为生机，化挑战为机遇，保持经济社会平稳较快发展。

　　今年政府工作的总体要求是：深入贯彻落实科学发展观，按照省委、省政府“三促进一保持”决策部署，把保增长作为首要任务，把实施《珠江三角洲地区改革发展规划纲要》作为保增长的重要契机，把扩大内需作为保增长的根本途径，把加快发展方式转变和结构调整作为保增长主攻方向，把深化重点领域和关键环节改革、提高对外开放水平作为保增长的强大动力，把改善民生作为保增长的出发点和落脚点，全力实施“工业立市、港口兴市、生态建市”发展战略，全力建设大型钢铁基地，全力建设重大基础设施，加快建设城乡协调、生态文明的科学发展试点市，推动经济社会又好又快发展，为实现湛江后发崛起奠定坚实基础。

　　今年经济社会发展的主要预期目标是：全市生产总值增长9%，其中第一产业增长5%，第二产业增长10%（工业增加值增长10%），第三产业增长10.5%；人均生产总值增长8%；单位生产总值能耗下降2.75%；全社会固定资产投资增长18%；港口货物吞吐量1.1亿吨；社会消费品零售总额增长15%；居民消费价格指数涨幅控制在4%左右；外贸进出口总额增长10%，其中出口增长8%，实际吸收外资与上年基本持平；地方财政一般预算收入增长10%，人均财政收入增长8.5%；城镇居民人均可支配收入增长8%，农村居民人均纯收入增长7%；城镇登记失业率控制在3.5%以内；人口自然增长率控制在8.1‰以下。

　　今年的主要任务和工作重点是：

　　一、强化工业立市，构建现代产业体系

　　今年是我市重大项目建设年，年内安排重点建设项目60个，计划完成投资124.8亿元，其中工业项目计划完成投资53亿元。必须牢牢把握工业立市不动摇，以工业化为核心调整优化产业结构，加快建设以先进制造业和现代服务业为重点的现代产业体系。

　　全力建设湛江钢铁基地项目。坚持把千万吨级钢铁基地项目作为全市经济工作的重中之重，举全市之力推进建设。加快项目征地拆迁和安置补偿工作，确保3月底前完成征地拆迁任务，建成搬迁村民过渡安置房并动迁入住。年内建成为钢铁基地直接配套的龙腾物流500万吨球团项目。全面推进钢铁基地外围配套基础设施建设，年内建成钢铁项目起步码头；动工建设茂湛铁路、东海岛铁路、海大路口至蔚律港疏港公路及东海岛跨海大桥、鉴江供水枢纽工程，推进钢铁基地配套系列码头前期工作。高起点、高标准完成编制钢铁产业发展规划，适时开展钢铁产业专题招商活动，及早谋划引进一批关联度高、投资量大、带动性强的配套项目，建设产业链完整、规模效益显著、辐射带动能力强、生态良好的钢铁产业基地。

　　着力培植工业增长新载体。以经济技术开发区东海岛新区和产业转移工业园区为重点，加快平整土地和完善基础设施，加大招商引资和项目建设力度，主动加强与广州市协作联动，建设广州（湛江）省级示范性产业转移工业园；完善龙岗（吴川）、顺德（廉江）省级产业转移工业园基础设施，提升园区功能，吸引更多项目落户建设。支持县（市）及湛江农垦筹建产业转移工业园和中心镇、专业镇兴办工业集中发展区。

　　促进中小企业平稳健康发展。全面落实国家、省支持中小企业健康发展和鼓励发展民营经济的政策措施，优化中小企业和民营经济发展环境。加大对中小企业的财税金融支持，健全融资担保体系，积极开展股权出质登记，扩大动产抵押范围，实施小额贷款公司试点。市财政安排500万元设立扶持中小企业发展专项资金，提供贷款贴息服务；对企业堤围费减半征收。实施“中小企业成长工程”，支持中小企业与大企业配套发展，加快转型升级。鼓励引导在外发展的本土企业家回乡投资创业。评选表彰百家成长型工业企业。

　　加速发展现代服务业。围绕钢铁、石化、家电、农海产品加工等重点产业，提供产前、产中、产后、研发等一条龙服务，促进制造业与服务业加快融合、互动发展。引导和推动企业通过管理创新和业务流程再造，逐步将发展重点集中于技术研发、市场拓展和品牌运作。完善科技开发、产品设计、工程设计、环境监测等科技服务。加快建设区域金融中心，改善和优化区域金融生态环境，吸引有实力的金融机构进驻湛江，壮大银行、资本、保险三大市场。建设一批枢纽型现代物流园区、配送中心。增加航线航班，提高空港客货流量。积极发展中介服务业及会展业。

　　加速发展商贸流通业。结合新城区开发和旧城区改造，推进建设赤坎旧大天然片、霞海港区片、经济技术开发区中央商务区和湛江水产城等，续建或动工建设一批标志性写字楼、高星级酒店，形成若干具有较大规模和较强带动力的现代服务业集聚区。建设海田批发市场等一批大型专业市场和县（市）、镇果蔬批发市场，培育一批商贸龙头企业，发展总部经济、连锁经营。扩大文化、体育、健康等服务性消费。落实国家促进房地产市场健康发展政策，扩大保障性住房供给，鼓励使用住房公积金购房，扩大住房消费。加强农村、城镇、社区商业网点建设，继续实施“万村千乡”市场工程，落实好“家电下乡”等消费政策，扩大农村地区消费。

　　加速发展特色旅游业。修编完善湖光岩大旅游区和东海岛旅游度假区等发展规划，支持湖光岩旅游区创建国家5A级旅游景区、东海岛旅游度假区创建国家4A级旅游景区，建设市区游客集散中心、霞山渔人码头、旅游码头。积极开发特色海岛旅游，推进滨海休闲旅游项目建设，建成特呈岛度假村。办好内容丰富、形式多样的旅游文化体育节庆活动。加强与琼北、两广九市、泛珠三角和广东中旅等旅游合作，共同开拓区域旅游市场。

　　推进自主创新工程。全面落实《湛江市自主创新行动计划》，力争年内实施100项以上科技计划项目。继续实施品牌带动战略，促进信息化与工业化融合，建设一批高新技术和先进适用技术项目，高新技术产业总产值增长10%以上。推动建设以广东海洋大学为依托的广东省海洋开发研究院。建立一批特色产业基地、工程技术和企业技术开发中心、农业技术创新中心，进一步推动企业成为自主创新主体。实施国家和省知识产权战略纲要，保持专利申请量和授权量继续增长。科技对工业经济增长贡献率达到48%。

　　二、强化以港兴市，增强港口龙头带动作用

　　深水大港是湛江后发崛起的强大引擎，必须推动港口从单一运输枢纽向资源配置枢纽提升，从单一港口运输向港口经济提升，实现港口、临港产业、现代物流和城市建设“四位一体”同步推进，增强大西南及中部地区重要出海通道和我市经济发展的龙头作用。

　　完善提升港口功能。从今年起三年内投入124亿元，其中年内投资30亿元，实施港口6大重点建设项目，全力推进集装箱、矿石、油品等专业化码头和综合性港区建设。完成霞山港区成品油码头建设和调顺岛港区泊位改造工程；加快宝满集装箱码头一期、东海岛港区杂货码头、霞山港区大型散货码头等项目建设；力促宝钢重件、砂石码头和东海岛大型油码头早日动工。实施雷州半岛县域港口群发展规划，推动重组徐闻港口资源，重点建设徐闻港区和雷州流沙港。

　　做大做强临港产业。依托港口和骨干企业优势，重点支持发展石化、能源、造纸和农海资源加工等产业，拉长壮大产业链，构建蓝色产业集群。加快建设临港石化基地，全力支持中石化、中石油、中海油等在湛企业发展壮大，年内确保东兴500万吨炼油配套完善项目投产，海洋油气当量达到1300万立方米；推进中海油300万吨沥青等项目前期工作。加快建设能源产业基地，力争徐闻洋前风电项目年内投产；动工建设奥里油发电厂油改煤、徐闻勇士风电场和湛江生物质发电项目；推进大唐雷州电厂、吴川吴阳风电场、中航油150万立方米储备库、湛江（廉江）国家石油储备基地和中海油液化天然气接收站等项目前期工作。加快建设林浆纸产业基地，着重推进70万吨木浆项目。加快建设农海资源加工产业基地，推动企业重组联合，提高集中度，促进产业上规模、上水平。

　　建设区域性航运中心和物流中心。完善港口货物运输配套设施，开辟东南亚近洋班轮航线和国内干线。加快推进铁矿石配送中心、配煤中心、储备油库和粮食、食用油、农资仓储等港口物流项目，发展石油、矿石、煤炭、粮食、化肥5大临港物流基地。创新港口经营模式，吸引业主兴建贸易、仓储、进出口产品加工和集散设施，吸引大宗散货向湛江港集聚。调整临港工业园功能，积极引进国内外大型物流企业和项目，申报建设宝满保税物流中心。制定《促进航运业发展的若干意见》，发展航运及船舶修造业。

　　增强湛江港（集团）股份有限公司龙头带动作用。推动湛江港（集团）股份有限公司规范运作，加快上市融资步伐；采用先进设备、技术和管理手段，促进港口设施、装卸工艺、运作手段现代化，完善港口服务功能，提升服务水平，努力提供比兄弟港口更独特、更具效率、更优质的服务，建成设施先进、管理科学、运行高效的现代化港口集团。

　　三、强化生态建市，打造绿色宜居湛江

　　良好的生态环境是湛江得天独厚的后发优势，必须把生态建市、绿色发展作为我市后发崛起的重要特色，贯穿于生产建设、生活消费各个方面，确保蓝天长在、碧水长流、空气长新、资源永续利用。

　　加强生态环境建设。坚持资源开发、工程建设与生态保护建设同步设计、同步施工、同步验收，从源头上控制污染和保护资源环境。修编《城市绿地系统规划》，年内建成滨湖公园，加快森林公园等改造和赤坎江景观整治二期工程，动工建设海湾大桥桥东公园、银帆公园和金沙湾观海长廊三期工程，结合市政道路建设改造扩大路树面积，确保城市绿地不减，绿化覆盖率不降。推进沿海防护林及重点林业生态工程，构筑沿海绿色生态屏障。深入创建国家卫生城市、国家环保模范城市、国家生态园林城市和林业生态市，加大名山景点、古树名木、海湾滩涂、古镇、古村落和古民居保护力度，扎实推进生态示范村、镇创建活动。继续实施“城乡清洁工程”，健全长效机制，不断巩固和发展已有成效。统筹解决人口问题，努力完成人口控制目标，实现人口与资源环境相协调。

　　推进资源节约和综合利用。落实最严格的耕地保护和节约用地制度。制定实施《投资项目用地管理办法》，确保土地供应向产业带动力强、经济效益好、资源能源消耗少的项目倾斜，促进土地集约高效利用。全面落实省全民节能行动实施方案，严格执行节能问责制，完善单位GDP能耗统计、监测和考核体系，实行重点企业能源审计和能源利用状况公告制度。着力抓好电力、石化、建材、纺织印染、造纸、制糖等行业和100家重点耗能企业节能考核，有序淘汰落后产能。推进建筑节能示范工程，实现城市新建筑物100%符合节能标准。大力推广农业节水灌溉技术，推行城镇生活用水阶梯收费，建设节水型城市。

　　严格控制污染物排放。实行主要污染物排放总量控制，建成国控、省控重点污染源在线监控系统，推行排污许可证、企业环境信用和污染责任保险制度，对重点环境问题和重点污染企业实行挂牌督办，确保完成国家和省下达的约束性指标。加强湛江港湾环境保护，突出抓好滨海污染治理。实施公共交通清洁能源改造，逐步实现市区公交车全部改用液化天然气。深入整治机动车废气超标排放。年内完成市区生活垃圾无害化处理、赤坎和霞山污水处理厂二期、麻章污水处理厂一期和东新渠整治等工程，确保县（市）城区全部建成污水处理厂；动工建设坡头和东海岛污水处理厂。建成和改造一批市区生活垃圾中转站。全面开征、合理调整污水处理费。筹建市区生活垃圾焚烧发电厂。加快县（市）城镇生活垃圾无害化处理场建设。

　　大力发展循环经济。实施《湛江市发展循环经济试点实施方案》，创建减量化、再利用、资源化生态产业园区，鼓励企业围绕“三废”回收和再利用，形成再生资源循环链；培育具有龙头带动和示范作用的循环经济企业，形成一批循环经济群体。实施一批风力、压差发电和水资源再生利用等循环经济示范项目，推进15家循环经济试点单位建设。实施循环经济百村计划，发展农村循环经济产业。

　　四、强化“三农”基础，统筹城乡发展

　　全面贯彻党的十七届三中全会和今年中央一号文件精神，坚持以增加农民收入、增进农民福祉为核心，努力开创农业和农村科学发展新局面，进一步强化我市现代农业在全省的领先地位。

　　加快发展现代农业。在稳定粮食生产基础上，因地制宜建设规模相对集中、市场相对统一的优势农产品生产基地，集中抓好2个国家级、48个省级名牌农产品、41个有机食品和95个绿色食品标准化生产，打响“半岛热带农业”品牌。扶持发展“一村一品”，加快特色农业强镇强村建设。大力发展现代渔业，推广无公害健康养殖，建立出口水产品加工原料安全示范区，振兴“南珠”产业。建设区域畜禽产销基地。加强与湛江农垦全方位战略合作，共同谋划发展现代农业、工业园区、物流商贸、垦区城市化和休闲旅游业等，支持农垦糖业上市融资，加快建设湛江花卉科技园，实现地方与农垦的优势互补、互利双赢。抢抓两岸关系和平发展机遇加强湛台农业合作，推进建设海峡两岸农业合作试验区。全面开展集体林权制度改革，完成造林24万亩。

　　推进农业产业化经营。采取贷款贴息、项目补助和投资参股等办法，支持农业龙头企业发展壮大，鼓励具备条件的争取上市融资。构建新型农村市场流通网络，沿徐闻、麻章、廉江一线布局农产品批发市场和集散中心，沿国道、省道乡镇兴建农村超市，在中心村和农民社区建设“农家店”，发展农产品连锁经营。年内建立健全行政村和村民小组两级集体经济组织，完成组织成员身份界定和集体资产确权登记，建立组织成员和股权档案管理制度；大力支持农村集体所有资产股份制改造，壮大农村集体经济。

　　完善农村土地承包经营权流转制度。落实中央有关政策，制定实施农村土地流转管理办法，推动土地适度规模经营。培育发展县、镇、村三级流转服务中介组织，完善农村土地流转市场体系。积极为土地流转创造良好条件，将合法土地承包经营权证书全部发放到户。

　　改善农村生产生活条件。安排农业和水利项目投资22亿元，重点建设雷州青年运河灌区改造、雷州半岛扩大治旱、水利防灾减灾、节水灌溉、人工增雨试验基地等项目，年内完成徐闻、遂溪北部湾海堤等12宗堤防修复加固。加快推进农村饮水安全工程，确保2011年前解决全市256.8万人饮水安全问题。实施县级以上现代标准农田示范区和国家农业综合开发土地治理项目，继续推进农村信息化、综合信息服务国家试点市工作。全面完成镇通行政村硬底化公路建设任务，行政村客运通达率95%。建设硇洲、乌石国家级中心渔港和龙头沙、草潭、海安国家一级渔港。

　　巩固和扩大新农村建设成果。坚持以点带面、整体推进，以实施“万村百镇”整治工程为载体，以“四通五改六进村”为主要内容，抓好35个生态文明区（片、带）建设；支持条件较好的地区创建生态文明镇，并将农场场部纳入创建范围，推动生态文明村连线扩面、集群发展。实施农村民居地震安全工程示范村建设。年内完成800个村庄新农村建设规划编制。支持特色鲜明的生态文明村发展休闲旅游产业，扩大效应。

　　五、强化投资拉动，加快基础设施和城市建设

　　充分发挥投资创造供给和需求的双重效应，以基础设施和城市建设为切入点，千方百计扩大投资，拉动内需，增强发展后劲。

　　从战略高度规划构建大湛江格局。按照“东拓、西扩、南展、北延”的城市发展思路，结合海滨大道改造，把经济技术开发区老区作为城市中心精品片区加快建设；完成编制《“一湾两岸”景观规划》，着力开发建设海东新城区，形成环海型城市特色；抓住湛江钢铁基地建设的机遇，完成修编东海岛分区规划，把东海岛建成工业发达、生态文明、宜业宜居的现代化钢铁新城；结合打造全国最大的热带花卉基地，规划建设集商住、观光、休闲为一体的新型生态城区。启动全市国民经济和社会发展“十二五”规划编制前期工作。完成《湛江市城乡发展战略规划》、《湛江市生态城市建设规划》、《湛江市科技发展战略规划》、《湛江市城市综合交通专项规划》以及麻章区、南三岛分区等规划编制和修编工作，开展科学填海造地、疏港大道两侧、旧大天然片区等控制性详细规划编制。争取《湛江市城市总体规划（2005-2020）》尽快获批实施。

　　加快城市基础设施建设。动工建设湛徐高速公路、海湾大桥西连接线一级公路、广湛高速公路官渡出入口立交改造等项目。加快建设国道325线文车至北罗坑一级公路和南三大桥。启动建设海东新区观海长廊、游艇俱乐部、城区防洪堤围工程。年内建成体育北路西段，完成人民大道、海淀路、北站路、站前路和海田片道路改扩建，加快疏港大道二期工程建设，启动海滨大道、奋勇大道、新湖大道、滨湖路二期、南方路片改扩建和整治。开展湛江大道前期工作。

　　积极推动旧城区和城中村改造。制定实施旧城区、旧厂场、旧村庄（城中村）改造管理规定、城市规划区内城中村村民建房管理办法和城乡结合部、城市交通主干道两侧、城中村宅基地建设管理办法。结合水产品批发市场搬迁和渔人码头建设，推进霞山区汉口路、洪屋路片区旧城改造，务求实质性进展。完成赤坎“三民路”修复工程，恢复古商埠面貌。继续抓好文保北村等城中村、城郊村和小街小巷改造。

　　优先发展城市公共交通。加快实施《湛江市城区公交发展规划（2006-2020年）》，增加公共财政投入，开拓多元化投资渠道，鼓励民间资本、境外资本以合资、合作等方式参与城市公共交通建设和运营。增开火车西站和市区出入口公交线路，完善海田公交总站、霞山汽车南站、湖光岩汽车客运站、麻章公交站及加气站，建设一批港湾式候车亭。开展城际轨道交通研究论证。

　　全面提高城市管理水平。努力推动城市管理从“突击型”向“规范型”转变、从“粗放型”到“精细化”提升、从“小城管”向“大城管”迈进。完善“二级政府、三级管理、四级网络”机制，全面推行城管执法重心下移，明确区、街责任和权利。建设数字化城市管理系统，开通“12319”城管服务热线。健全制度规范城市交通秩序，着力疏解市区节点堵车、塞车问题。

　　六、强化改革开放，激发科学发展活力

　　坚持改革开放主旋律，不断完善社会主义市场经济体制，不断提高开放型经济水平，为科学发展提供体制保障和强大动力。

　　深化国有资产管理和国有企业改革。完善出资人制度和国有资本经营预算制度，创新国有资产运营和监管模式。健全国有控股企业法人治理结构，加快建立现代企业制度，打造具有较强竞争力的市场主体。支持湛化股份公司等大型企业资本经营和上市融资，鼓励航运集团、汽运集团、自来水公司、海滨宾馆等企业引入战略投资者开展合资合作，推进与省属企业合作发展，积极盘活半球、三星等企业存量资产。加快市属国有劣势企业退出市场步伐。

　　完善公共财政制度。以基本公共服务均等化为取向，完善保障和改善民生的财政机制，构建绩效优先、保障有力、规范透明的财政支出管理体系。逐步推行财政专项资金竞争性分配。健全市、县（市、区）、乡镇三级财权与事权相匹配的体制，调整完善市对区财政管理体制，逐步均衡市与区财力。建立统一的财政资金管理信息系统。坚持依法治税理财，加强税收征管，确保收入应收尽收。积极推进镇乡财政和村级财务管理制度改革。

　　加强投融资平台建设。充分用好当前国家积极的财政政策和适度宽松的货币政策，加大融资力度。积极推进政、银、企合作，拓展金融服务领域，推动成长型企业改制上市直接融资。加快市水务集团和市基础设施建设投资公司整体融资工作。支持市商业银行改革股权结构，实现跨区域稳健发展，努力打造现代化金融企业。深化农村信用社改革，积极创建村镇银行。采取政府主导、政策推动、市场化运作方式，推进城市土地资源商品化、存量资产资本化、无形资产有形化、市政投入社会化经营。

　　保持出口稳定增长。沉着应对国际金融危机，着力转变外贸发展方式，实施“科技兴贸”和“市场多元化”战略，优化出口商品和市场结构，提高出口产品质量和附加值。推进加工贸易上下游产业链配套建设，推动加工贸易企业转型升级。加强“大通关”口岸基础设施建设，创新海关、检验检疫监管模式，构建进出口“绿色通道”。鼓励企业到国外开办公司，进行商标注册、质量认证，促进海外投资。积极应对国际贸易壁垒和摩擦，注重防范市场风险。

　　提高吸收外资质量和水平。紧密围绕钢铁、石化、能源等主导产业招商引资，以世界500强企业和战略投资者为重点，主动承接国际重化产业、先进制造业、高新技术产业和现代服务业转移，着力引进一批产业链长、带动力强的龙头型、基地型项目。创新招商引资方式，加强重点园区和行业龙头企业招商，精心策划、包装、推介一批可操作性强的招商项目，落实招商配套条件，提高招商引资实效。支持现有外商投资企业增资扩产。认真抓好省第五届“山洽会”等招商引资活动签约项目的跟踪落实工作。

　　推进区域经济合作。充分发挥我市在北部湾地区的优势，更加积极参与北部湾经济区合作发展，重点在建设西南出海大通道、区域市场体系、相关相近产业链条构建、区域旅游、北部湾生态环境保护等方面扩大合作；举办北部湾城市论坛。务实推进与中国—东盟自由贸易区、大西南、港澳及珠三角地区的经济技术合作，尤其要抓住洛湛铁路即将开通的有利条件，搭建内陆“无水港”平台，拓展与中南地区的合作领域。以两岸直接“三通”为契机，深入推进湛台经贸合作。全面落实CEPA在我省先行先试的政策措施，推动湛港澳合作发展。提高与国内外友好城市合作成效。加快推进四川省汶川县龙溪乡地震灾区对口援建工作。

　　七、强化和谐发展，推进社会民生建设

　　坚持把社会民生建设作为构建和谐湛江的首要任务，着力解决涉及群众利益的难点热点问题，努力促进社会公平正义，维护社会稳定，让发展成果更多地惠及最广大群众。

　　努力办群众满意的教育。抓好以聚集办学为主要内容的中小学校布局调整试点，实行优质学校与薄弱学校捆绑办学，推进规范化学校建设，促进义务教育均衡发展。大力普及高中阶段教育，毛入学率达到71.5%。启动市职业教育中心园区建设，组建职业教育集团。积极稳妥解决中小学代课教师问题，扎实推进教师工资福利待遇“两相当”工作，实施义务教育教师绩效工资制度。推行师范毕业生到农村学校任教退费和顶岗实习制度。支持驻湛高校和民办教育发展，推进广东医学院新校区建设前期工作。

　　大力发展文化体育事业。加强公共文化设施建设，建成湛江粤剧院，动工建设市档案新馆；完成市区数字电视整体转换；开展市演艺中心、文化艺术中心、书画院和报业新闻大厦前期工作；把乡镇文化站建成基层文化信息资源共享服务网点，推动乡镇文化中心全部达标入级。实施第三次文物普查，积极申报全国、全省非物质文化遗产保护项目。建设湛江跳水训练馆和游泳跳水馆，打造国家跳水训练基地；举办市第十一届运动会、第十届体育节、第二届红土文化艺术节、第三届国际海上龙舟邀请赛等赛事活动，参加全国第十一届运动会。

　　提高医疗卫生服务水平。积极推进医药卫生体制改革，扎实开展医疗服务和药品价格改革试点工作，完善平价医疗服务体系，为群众提供安全、有效、便捷、廉价的医疗服务。调整职工医保政策，降低起付标准和个人自付比例，提高异地住院报销标准，缩短医保待遇等待期，减轻参保职工就医费用负担。组建市紧急救援中心。继续改造乡镇薄弱卫生院，基本完成乡镇卫生院信息化建设。加强社区卫生服务站建设。着力做好重大传染病防控和卫生监督执法工作。积极推进中医强市建设，逐步把市第一中医院建成现代化综合性中医院、市第二中医院建成中西医结合医院。

　　保障人民群众安居乐业。健全自然灾害、公共卫生、公共安全等突发事件应急管理机制。加强市场价格监测和监管，维护价格秩序和总水平基本稳定。推进实施食品药品放心工程，深入开展食品药品安全专项整治。坚持信访维稳工作责任考核、领导干部基层大接访和包案制度，完善农村维稳体系，加强矛盾纠纷调处与疏导，力争涉法涉诉信访案件办结率85%以上，息诉罢访率85%以上，越级上访、集体上访持续下降。加快推进国防综合基地和民兵基层组织基本建设，完善双拥优抚安置政策制度，巩固发展军政军民团结。健全消防安全保障体系，多管齐下排查整治安全隐患，完成安全生产控制考核指标任务。强化社会治安防控体系建设，依法严厉打击“两抢一盗”等严重犯罪和黑恶势力，保持打击走私高压态势，完善反恐工作机制。进一步抓好外事、侨务、民族宗教、统计、人事、对台、人防、地震、修志、老区以及妇女儿童、残疾人、老龄等工作。

　　着力办好15件民生实事。完成20户以上已通电自然村广播电视“村村通”任务；建成200间“农家书屋”；落实积极就业政策，大力开发社区就业岗位和公益性岗位，新增城镇就业岗位5万个；实施创业带动就业工程，组织创业培训2000人，带动1万人就业；抓好登记失业人员、大中专毕业生职业技能培训，完善就业援助制度，实现下岗失业人员就业4万人，其中困难群众就业2000人；组织农村劳动力免费技能培训5万人，实现转移就业16万人；开展送岗位活动，举办200场农民工招聘会；把农村家庭年人均纯收入低于1500元的困难户纳入低保范围；继续扩大社会保险覆盖面，妥善解决被征地农民养老保险问题，完善城乡社会救助体系，年内企业养老保险达到60.8万人、失业保险达到32.7万人、医疗保险达到46.2万人、工伤保险达到25.5万人、生育保险达到33.1万人以上；建设一批经济适用住房和廉租房，逐步解决市区城镇低收入家庭住房困难；继续推进农村困难群众茅草房、泥砖房和危旧房改造工程，年内完成2万户改造任务；建设水库移民新房4500户，为18500名水库移民解决住房困难；新建村级敬老院120间，为2500多名农村五保老人解决住房困难；按“规划到户、责任到人”要求，抓好第二批整村推进扶贫开发20个村庄试点，实现脱贫目标；推进产业化扶贫，重点扶持7家省级扶贫农业龙头企业，带动1万贫困户脱贫致富。

　　八、强化政府建设，提高领导科学发展能力

　　按照为民、务实、清廉的要求，以转变职能、增强决策力、执行力和公信力为重点，进一步推动政府管理创新，提高行政效能和服务水平，为实现湛江科学发展、跨越发展提供坚强保证。

　　依法行政，建设法治政府。按照国务院《关于加强市县政府依法行政的决定》要求，完善行政决策规则和程序、重大行政决策听证和合法性审查、规范性文件定期清理公布、规范行政自由裁量权等配套制度，进一步梳理划清各级行政部门的权责范围，严格按照法定权限和程序行使权力、履行职责。加强行政复议和行政应诉工作，强化行政执法评议考核。自觉接受人大及其常委会的监督，按规定报告工作；主动听取政协和各民主党派、人民团体的意见；积极办好人大代表议案、建议和政协委员提案。

　　科学理政，建设责任政府。推进新一轮政府机构改革，进一步优化政府组织体系。健全工作量化考核办法，开展政府工作绩效评估，强化工作责任目标刚性要求，建立体现唯一性的行政问责制度。全面实施领导干部落实科学发展观评价指标体系及考核评价细则，出台《市直科级及以下公务员年度考核办法》，公布年度考核结果，强化考核结果运用。今年是我市转变作风落实年，要大力营造实干有为、敢于负责、善于落实的浓厚氛围，加快建立高效快捷、迅速果断的执行机制，确保每个项目、每项工作定一件、干一件、成一件。

　　为民执政，建设服务政府。开展第四轮行政审批制度改革。加强行政服务中心建设，建立市、区两级政府审批联动以及市行政服务中心与各职能部门、区级政府“三位一体”联动工作机制，并延伸到重点乡镇。加快行政审批网络平台建设，实现市级所有审批事项网上申报和审批。健全行政投诉处理、政务信息资源共享、重大投资项目、行政绩效测评四大电子监察系统。大力推进政务公开，完善政府信息发布制度，提高政府工作透明度和公信力。

　　从严治政，建设廉洁政府。认真落实党风廉政建设责任制，着力构建惩治和预防腐败体系，进一步完善和健全工程招投标、土地出让、产权交易、政府采购、涉农专项资金使用、财政性投资项目管理制度，加大领导干部经济责任和专项资金审计力度，多种形式向社会公开审计结果。严肃查处腐败行为，坚决纠正损害群众利益的不正之风。力戒“文山会海”，克服形式主义、官僚主义，市政府要争取减少四分之一的会议和文件。坚持艰苦奋斗、勤俭节约，严格控制党政机关一般性行政经费支出。政府机关每一位公务员都要牢固树立根本宗旨意识，全心全意为民，克己奉公从政，努力建设团结开拓、求真务实、亲民为民、勤政廉政的政府。

　　各位代表，我市改革开放和现代化建设正在进入一个新的重要历史时期，在新的起点上加快实现湛江后发崛起，是时代赋予我们的光荣使命，是全市人民对我们的殷切期望，是我们必须履行的神圣职责。让我们在中共湛江市委的正确领导下，全面落实科学发展观，解放思想、坚定信心、振奋精神、奋力开拓、扎实工作，坚定不移地在科学发展的新征途上阔步前进，为加快建设城乡协调、生态文明的科学发展试点市而努力奋斗！
